# Synthesized 18-Lead Electrocardiogram in Diagnosing Posterior Stemi-Equivalent Acute Coronary Syndrome in Patients with NSTEMI

**DOI:** 10.1155/2022/9582174

**Published:** 2022-08-17

**Authors:** Tomoki Horie, Rikuta Hamaya, Tomoyo Sugiyama, Hidenori Hirano, Masahiro Hoshino, Yoshihisa Kanaji, Tetsumin Lee, Taishi Yonetsu, Tetsuo Sasano, Tsunekazu Kakuta

**Affiliations:** ^1^Department of Cardiology, Tsuchiura Kyodo General Hospital, Tsuchiura, Japan; ^2^Division of Preventive Medicine, Department of Medicine, Brigham and Women's Hospital, Harvard Medical School, Boston, MA, USA; ^3^Department of Epidemiology, Harvard T.H. Chan School of Public Health, Boston, MA, USA; ^4^Department of Cardiovascular Medicine, Tokyo Medical and Dental University (TMDU), Tokyo, Japan

## Abstract

**Objective:**

To assess the clinical utility of synthesized V7–V9 ST-segment elevation (sV7-9 STE) in patients with 12-lead-electrocardiogram (ECG)-based non-STE myocardial infarction (NSTEMI) in diagnosing left circumflex artery (LCx) STEMI-equivalent acute coronary syndrome (ACS).

**Background:**

The 12-lead-ECG is insufficient for diagnosing patients with ACS, especially those with an LCx culprit.

**Methods:**

We retrospectively examined 219 patients with NSTEMI who underwent synthesized 18-lead ECG acquisition on admission and urgent catheterization. Associations between baseline variables, including sV7-9 STE and LCx STEMI-equivalent ACS, were analyzed using logistic regression models and receiver operating characteristics. LCx-culprit ACS was defined as thrombolysis in myocardial infarction (TIMI) 0–1 flow. The association between sV7-9 STE and myocardial damage was also assessed.

**Results:**

The mean (SD) age of the population was 68.8 (12.0) years, and 81.7% were men. LCx-culprit NSTEMI occurred in 58 (26.5%) patients and 15 (6.8%) were LCx STEMI-equivalent. SV7-9 STE was observed in 16 patients (7.9%). SV7-9 STE was the sole significant predictor of LCx STEMI-equivalent ACS with an odds ratio of 19.0 (95% CI: 5.6–63.9, *p* < 0.001), area under the curve of 0.71 (95% CI: 0.58–0.84), sensitivity of 46.7%, and specificity of 95.6%. After adjustment for confounders, sV7-9 STE was significantly associated with a 308% (95% CI: 78–834%) increase in peak high-sensitivity cardiac troponin I (*p*=0.001).

**Conclusions:**

SV7-9 STE had sole preprocedural diagnostic utility in detecting LCx STEMI-equivalent ACS with greater myocardial damage among patients with 12 ECG-based NSTEMI. The use of synthesized extra leads on admission may help identify patients with NSTEMI requiring primary revascularization.

## 1. Introduction

Standard 12-lead electrocardiography (ECG) is essential for diagnosing ST-segment elevation myocardial infarction (STEMI) [1, 2]. A major limitation of the 12-lead ECG is its inability to diagnose STEMI with a left circumflex artery (LCx) culprit [3–5]. Such cases may be misdiagnosed as non-STEMI (NSTEMI), which may lead to delayed reperfusion [6]. Posterior wall STEMI-equivalent acute coronary syndrome (ACS) is likely to be underdiagnosed because a longer distance between the lateral leads and the ventricular wall can cause lower recorded voltages and because no lead directly covers the posterior left ventricular wall. The European Society of Cardiology and American Heart Association/American College of Cardiology guidelines recommend acquiring posterior leads V7 through V9 for suspected cases of acute LCx occlusion, such as in patients with typical symptoms and an initially nondiagnostic ECG or ST-segment depression (STD) in leads V1-3 [1, 2, 7]. However, acquisition of posterior leads V7-9 requires meticulous reattachment from the chest to the back and is yet to be adopted in routine clinical practice [8–10].

A synthesized 18-lead ECG is a specialized cardiograph that mathematically computes the virtual waveforms of the right-sided (V3R, V4R, and V5R) and posterior leads (V7, V8, and V9) based on a standard 12-lead ECG input. Computation is immediate and offers a pragmatic solution to the difficulties in acquiring true posterior leads. Several studies have reported the clinical usefulness of synthesized 18-lead ECG, including prediction of the origin of atrial and ventricular arrhythmias [11, 12]. However, whether additional consideration of the synthesized leads could facilitate improved detection of the acute coronary syndrome (ACS) requiring primary revascularization, particularly in patients with LCx-culprit lesions diagnosed as NSTEMI, remains unknown [13].

Therefore, in the present study, we investigated the diagnostic utility of synthesized posterior leads in identifying those with STEMI-equivalent LCx-culprit ACS, defined as thrombolysis in myocardial infarction (TIMI) 0 to 1 flow, among patients with 12-ECG-based NSTEMI. We hypothesized that synthesized V7–V9 ST-segment elevation (sV7-9 STE) would be diagnostically useful for LCx STEMI-equivalent ACS in patients with greater myocardial damage.

## 2. Methods

### 2.1. Study Population

Institutional registry data of consecutive patients with NSTEMI who underwent urgent catheterization within 48 h of admission at Tsuchiura Kyodo General Hospital between April 2016 and September 2020 were analyzed. We included patients with an identifiable culprit vessel who underwent percutaneous coronary intervention (PCI). Patients who were not triaged at the emergency department after admission had only a paper recording of the 12-lead ECG, without synthesized 18-lead ECG, and were excluded. The following patients were also excluded: those with a history of cardiac artery bypass grafting surgery, symptom onset over 48 h before admission, abnormal QRS morphology including branch bundle blocks and artificial pacing, and chronic total occlusion, defined as a presumed TIMI 0 flow for longer than 3 months.

**Figure 1:**
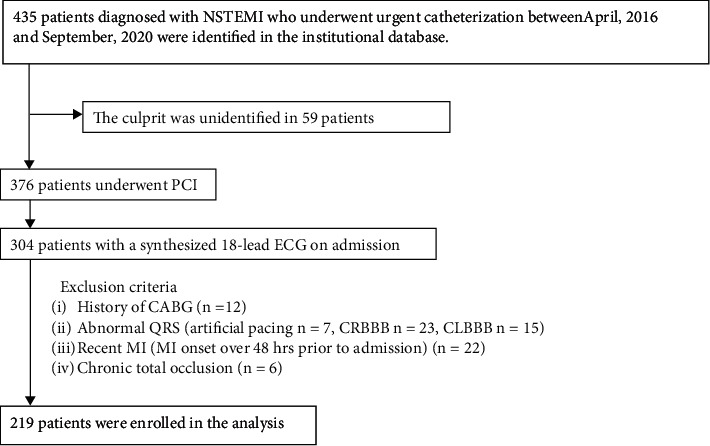
Study flow chart. 435 non-ST-segment elevation myocardial infarction (NSTEMI) patients were screened for eligibility. 376 patients underwent PCI. 304 patients had a recording of a synthesized 18-lead ECG on admission.85 patients were excluded according to the criteria. Finally, 219 patients were enrolled in the primary analysis. electrocardiogram, ECG; CRBBB, complete right branch bundle block; CLBBB, complete left branch bundle block; MI, myocardial infarction.

Of the 435 patients with NSTEMI screened for eligibility, 219 were enrolled in our primary analyses (Figure 1). The study was approved by the Institutional Ethical Review Board of Tsuchiura Kyodo General Hospital. The present study also complied with the Declaration of Helsinki for investigation in humans, and prior to enrollment, all patients provided written informed consent for institutional data registration and its utilization in future clinical research. Prompt optimal medical therapy was initiated in all patients before urgent PCI and guideline-directed medical therapy was continued throughout the study period.

### 2.2. Acquisition of ECG

The standard 12-lead ECG was digitally recorded on admission using a specialized electrocardiogram expanded with the synECi18 program (Nihon Kohden, Japan). Synthesized right-sided chest leads V3R, V4R, V5R, and posterior leads V7, V8, and V9 were readily computed from the 12-lead ECG and available using a dedicated viewer (Prime Vita Plus; Nihon Kohden, Japan). Briefly, the synthesized ECG leads were mathematically computed by calculating the electrical vector of the heart from the standard 12-leads and projecting this information onto the extra leads with previously obtained coefficients [8, 11]. Herein, the actual posterior V7-9 leads were recorded at the time of catheterization in approximately three-quarters of the total cohort (*n* = 164, 74.9%).

### 2.3. Assessment of ST-Segment Morphology

STE of ≥0.5 mm over the corresponding isoelectric line was considered significant in synthesized and actual leads V7–V9 [2, 7]. ST-segment morphology was otherwise categorized as showing no change, ST-segment depression (STD) if >0.5 mm, and inverted T-wave, if these changes were documented in at least two continuous sV7-V9 leads. Otherwise, the ST-segment morphology of the sV7-9 leads was considered unchanged. ECGs were analyzed by two independent cardiologists who were blinded to the clinical presentation, and a consensus reading was obtained in cases of discordance.

### 2.4. Coronary Angiography

Patients underwent urgent coronary angiography (CAG) with a guiding catheter via the transradial or transfemoral approach. Multivessel disease (MVD) was defined as stenosis of >70% of the diameter of at least two major vessels (≥2 mm in diameter) [14].The culprit lesion was diagnosed based collectively on CAG, electrocardiography, echocardiography, and the presence of intravascular thrombus in the appropriate cases. Diagnoses of the culprit lesions were established with the agreement of at least two expert cardiologists. The extent of coronary flow limitation was evaluated according to the TIMI flow grade [15].As our primary outcome, we defined LCx STEMI-equivalent ACS as LCx-culprit NSTEMI with TIMI 0 or 1 flow.

### 2.5. High-Sensitivity Cardiac Troponin-I

High-sensitivity cardiac troponin I (hs-cTnI) was used to diagnose and assess myocardial damage in NSTEMI (The Abbott Architect Troponin I assay; Abbott Diagnostics, Abbott Park, IL, USA). Biochemical measurements were performed at admission, before PCI, within one hour of PCI completion, 6, 12, 18, and 24 h after PCI, and at six-hour intervals thereafter if the values were still increasing. Peak hs-cTnI level was defined as the highest hs-cTnI level after admission.

### 2.6. Statistical Analysis

Categorical variables are presented as absolute frequencies and proportions and were compared using the chi-square or Fisher's exact tests. Continuous variables are expressed as mean ± standard deviation for normally distributed data and as median (interquartile range) for nonnormally distributed data. They were compared using Student's *t* test and the Mann–Whitney *U* test, respectively.

The primary analysis comprised univariate logistic regressions and receiver operating characteristic (ROC) curves to evaluate the associations between the following baseline patient characteristics and LCx-culprit NSTEMI with TIMI 0–1 flow: the presence of sV7-9 STE (yes/no), V1-3 STD (yes/no), age (continuous), sex (man or woman), left ventricular ejection fraction (%, continuous), Killip grade (1 or other), GRACE score (per 10 units, continuous), and hs-cTnI on admission (per 1000 ng/L, continuous). We also computed the area under the curve (AUC), sensitivity, specificity, and accuracy of the associations. Considering the effect of ECG on angiography time, we conducted a sensitivity analysis excluding 46 patients with an ECG-to-angiography time of over 12 hours.

Further, we evaluated the association between sV7-9 STE (yes/no) and peak hs-cTnI levels using multivariate linear regression analysis. Peak hs-cTnI was logarithmically transformed to approximate the normal distribution, and we interpreted exp (coefficient) − 1 as the percentage difference in mean peak hs-cTnI. The models were adjusted for V1-3 STD (yes/no), sex (male or female), GRACE score (per 10 units, continuous), and hs-cTnI on admission (per 1000 ng/L, continuous).

Agreement between the synthesized and actualV7-9 ST-segment morphologies was assessed using Cohen's kappa coefficient in patients who underwent both measurements. In these cases, the association between LCx-culprit NSTEMI with TIMI 0-1 flow and actual V7-9 STE, along with sV7-9 STE, was assessed using univariate logistic regression and ROC curves. The AUC, sensitivity, specificity, and accuracy of these associations were calculated and the correlation between two ROC curves, based on their AUCs, was tested using DeLong's test.

All analyses were conducted using R version 3.6.3 (The R Foundation for Statistical Computing, Vienna, Austria). Bonferroni method-adjusted two-sided *p* < 0.05 was considered significant.

## 3. Results

### 3.1. Study Population and Baseline Characteristics

A total of 219 patients with NSTEMI were included in this study, with a mean (SD) age of 68.8 (12.0) years and 179 men (81.7%). Sixteen patients (7.9%) presented with sV7-9 STE. Baseline patient characteristics according to sV7-9 STE occurrence are summarized in Table 1. Patients with sV7-9 STE were more likely to present with STD in V1-3, higher hs-cTnI, and lower GRACE risk score on admission, and less likely to present with V4-6 and inferior lead STD, compared with those without sV7-9 STE. In this cohort, none of the patients presented with isolated V6 STE or synthesized V7 STE.

Angiographic data are summarized in Table 2. During catheterization, LCx was diagnosed as the culprit's vessel in 58 (26.4%) patients. Most patients with sV7-9 STE were diagnosed with an LCx-culprit (left anterior descending artery (LAD), 1 (6.2%); LCx, 13 [81.2%]; right coronary artery (RCA),2 (12.5%)). A higher proportion of patients with sV7-9 STE had TIMI 0 or 1 culprit flow compared to those withoutsV7-9 STE (8 of 16 (50.0%) *vs*. 21of 203 (10.3%), respectively). The prevalence of MVD was similar between the two groups (5 (31.2%) *vs*. 56 (27.6%)).

### 3.2. Associations of Synthesized V7-9 STE and TIMI Flow-Defined LCx STEMI-Equivalent ACS

Fifty-eight patients were diagnosed with LCx-culprit NSTEMI, of which 15 patients had an occluded culprit of TIMI 0 or 1 flow. We diagnosed these patients with TIMI flow-defined LCx STEMI-equivalent ACS. Among these, sV7-9 STE was observed in 7 patients (46.7%). Synthesized V7, V8, and V9 STE were observed in six (40.0%), seven (46.7%), and seven (46.7%) patients, respectively, the prevalence of which differed from that in patients with LCx TIMI 2 or 3 flow (Supplemental Table 1). No other sV7-9 characteristics differed according to TIMI flow status.

**Figure 2:**
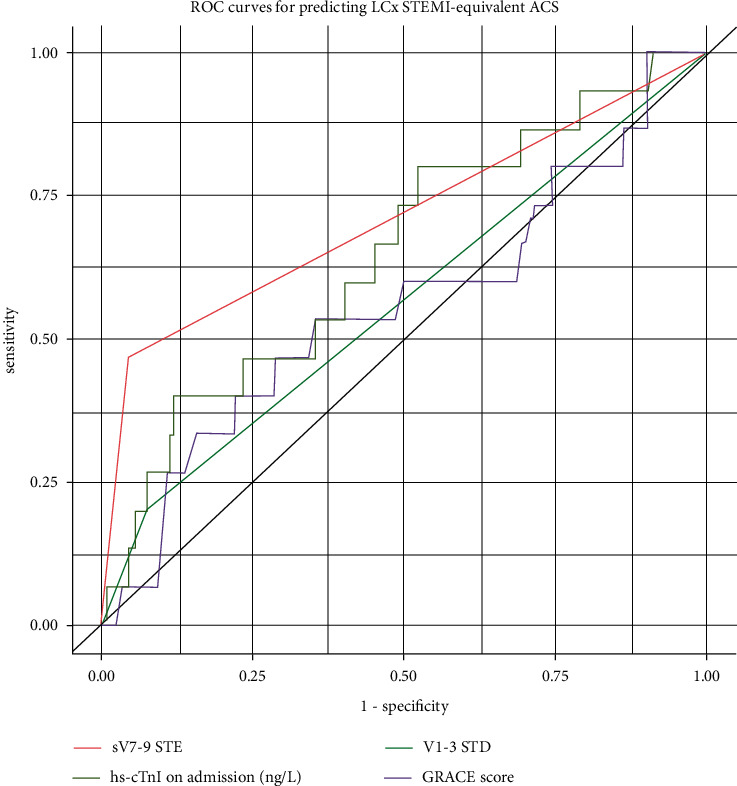
ROC curves for predicting LCx STEMI-equivalent ACS. Comparison of receiver operating characteristics (ROC) curves for typical baseline parameters. The top panel shows the ROC curve for diagnosing left circumflex (LCx) ST-segment elevation myocardial infarction (STEMI)-equivalent acute coronary syndrome (ACS) when defined by thrombolysis in myocardial infarction (TIMI) flow, in which sV7-9 STE showed the best diagnostic performance in terms of area under the curve (AUC).

In the logistic regression models, sV7-9 STE was a significant predictor of LCx STEMI-equivalent ACS among the baseline covariates, with an odds ratio (OR) of 19.0 (95% CI: 5.6–63.9, *p* < 0.001). According to the ROC analysis, the AUC was 0.71 (95%CI: 0.58–0.84), with a sensitivity of 46.7%, specificity of 95.6%, and diagnostic accuracy of 92.2% (Figure 2 and Table 3). No other baseline characteristics were comparable to those of LCx STEMI-equivalent ACS. This was supported by the results of the sensitivity analysis, which excluded patients with an ECG to angiography time of over 12 hours (Supplemental Table 2).

### 3.3. Associations of sV7-9 STE and Myocardial Damage

According to the multiple linear regression analysis in the total cohort, sV7-9 STE and hs-cTnI on admission were independently associated with peak hs-cTnI (308% (95% CI: 78–834%) with the presence of sV7-9 STE, *p* < 0.001, 11.1% (95% CI: 7.47–14.9%0 per 1000 ng/L increase in baseline hs-cTnI, *p* < 0.001) (Table 4).

### 3.4. Agreement between sV7-9 and Actual V7-9

The actual V7-9 leads were available at the time of catheterization in 164 (74.9%) patients. The STE of the actual V7-9 leads was observed in 13 patients. The actual and synthesized posterior leads exhibited excellent agreement in categorizing ST-segment morphology, with an overall kappa value of 0.85 (95% CI: 0.77–0.92) as shown in Supplemental Table 3. When limited to 164 patients, actual V7-9 STE was a significant predictor of LCx STEMI-equivalent ACS, with an OR of 12.9 (95% CI: 3.3–49.6). The AUC of actual and synthesized V7-9 STE were similar (0.68 *vs*. 0.72, respectively, *p*=0.317) (Supplementary Table 4).

### 3.5. Case Details

Details of the 16 patients with sV7-9 STE are provided in Supplemental Table 5. Thirteen patients were diagnosed with an LCx culprit, whereas one and two patients with sV7-9 STE had LAD and RCA culprits, respectively. One patient with an LAD culprit had sV7-9 STE, which did not change after revascularization, suggesting early repolarization with no diagnostic utility for ACS. Two patients with an RCA culprit had MVD. One patient had a history of myocardial infarction in the LCx, which might have affected the ST-segment morphology of the sV7-9. In another patient, the RCA distal branch was the culprit vessel, compatible with a posterior infarction. Seven of nine patients who underwent cardiac magnetic resonance imaging (MRI) showed late gadolinium enhancement (LGE) of the left ventricular posterior wall.

**Figure 3:**
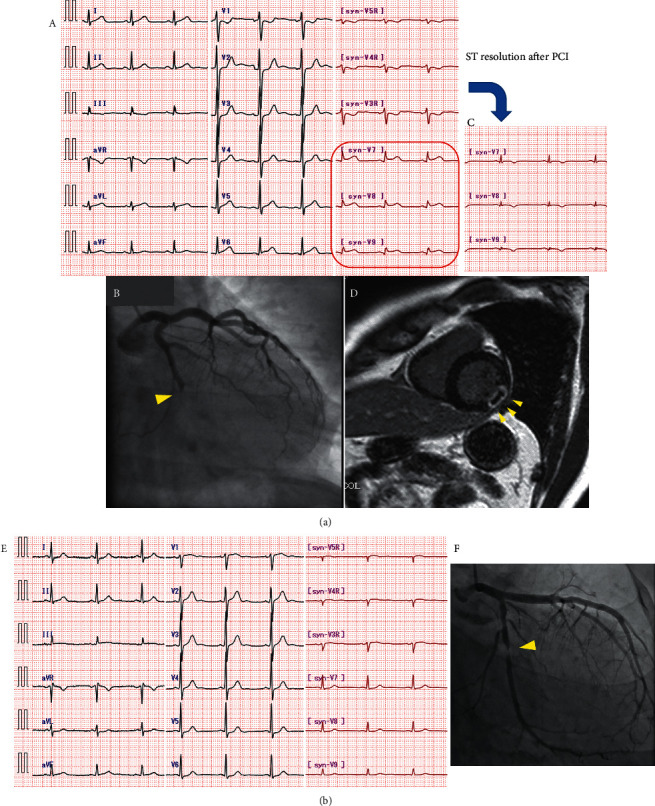
Representative cases. (a) The case is a 44-year-old man. On admission, synthesized V7-9 ST-segment elevation (STE) is observed, accompanied by ST-segment depression in V1 and V2 (A). Urgent coronary angiography confirmed thrombolysis in myocardial infarction (TIMI) 0 flow of the mid left circumflex (LCx) culprit (B). After successful revascularization, ST-segment resolution in synthesized V7-9 (C), and late gadolinium enhancement of the posterior wall (D) were observed. (b) The case is a 42-year-old man. On admission, the electrocardiogram including synthesized V7-9 was nondiagnostic (E). Urgent CAG revealed 90% stenosis with TIMI 3 grade flow of the mid LCx (G).

A representative case of sV7-9 STE and LCx STEMI-equivalent ACS is illustrated in Figure 3(a). A 44-year-old man presented with chest pain and an elevated arrival hs-cTnI level of 350 ng/L. On admission, ST-segment depression was observed in the anterior leads, accompanied by sV7-9 STE. Urgent coronary angiography confirmed TIMI 0 flow in the mid-LCx culprit vessel. After successful revascularization, ST-segment resolution was observed in the sV7-9, and cardiac MRI detected left ventricular posterior wall LGE consistent with myocardial damage of the posterior infarction.  Figure 3(b) shows another case of LCx-culprit NSTEMI, wherein the ECG including synthesized V7-9 was nondiagnostic, and coronary angiography revealed 90% stenosis with TIMI 3 flow.

## 4. Discussion

This is the largest study to investigate the clinical utility of sV7-9 leads in detecting LCx STEMI-equivalent ACS in patients with 12-lead ECG-defined NSTEMI. Our main findings were:1) sV7-9 STE was observed in 7.4% of patients with NSTEMI; 2) LCx-culprit NSTEMI comprised 26.4% of the total cohort; 3) sV-9 STE demonstrated the highest AUC and specificity in detecting LCx STEMI-equivalent ACS, defined as TIMI 0 or 1 flow; 4) sV7-9 STE was independently and significantly associated with a greater peak hs-cTnI; and 5) synthesized and actual posterior leads showed excellent agreement in categorizing ST-segment morphology with similar diagnostic abilities. Thus, synthesized extra lead findings on admission may help identify patients with NSTEMI requiring primary revascularization.

LCx is overrepresented as the culprit of ACS in the NSTEMI population than in the STEMI population [16]. Hishikari et al. [17] used intracoronary ECG and reported that among patients with NSTEMI, intracoronary ECG STE was observed significantly more often in the LCx, suggesting underdiagnosis of STEMI-equivalent patients with an LCx culprit. We believe that synthesized 18-lead ECG potentially enables proper diagnosis in some of these patients. The greatest appeal of synthesized 18-lead ECG is its ability to immediately provide virtual waveforms of right-sided chest leads (V3R, V4R, and V5R) and back leads (V7, V8, and V9) from standard 12-lead ECG information with only the click of a button. Actual and sV7-9 waveforms are reportedly almost identical [8, 10], concordant with our observations. In the one case with sV7-9 STE without STE in the actual V7-9, synthesized posterior leads were acquired 24 h before actual posterior leads, which may have changed the ST-segment morphology. Owing to its simplicity and reliability, synthesized 18-lead ECG is easily accessible by any clinician, including those without expertise in cardiology and those at clinics or other small facilities. Notably, despite its high specificity, the sensitivity of sV7-9 STE for diagnosing TIMI flow-defined LCx STEMI-equivalent ACS remained low at 46.7%. This is largely due to the low prevalence of sV7-9 STE. In clinical practice, diagnosing patients with a potentially high risk of STEMI-equivalent ACS is the priority, wherein synthesized V7-9 could be meritorious.

We observed a strong association between sV7-9 STE and the peak hs-cTnI, which could be used to estimate the prognosis of patients with ACS [18]. Wang et al. [19] evaluated 1,957 patients with NSTE-ACS, of which 528 (37%) had an occluded culprit artery on angiography, with a greater proportion of arterial occlusion in the inferolateral territory than in the anterior territory (*n* = 331 *vs*. 197, respectively). Patients with an occluded culprit artery had larger infarcts and a higher risk-adjusted 6-month mortality. Bahrmann et al. [20] studied 448 consecutive patients with NSTEMI who underwent early coronary angiography within 72 h of symptom onset, among which 130 (29%) had an occluded coronary artery mainly in the vessels supplying the inferolateral or posterolateral myocardium. These patients had more risk-adjusted 6-month clinical events than those without an occluded artery. Not only did we show a clear association between sV7-9 STE and higher peak hs-cTnI in the entire cohort, but we also observed a comparably higher peak hs-cTnI among patients with sV7-9 STE when limited to LCX STEMI-equivalent ACS. Therefore, the use of sV7-9 information could facilitate better triage of patients with 12 ECG-based NSTEMI who need emergent revascularization. This would help prevent the detrimental consequences of delayed reperfusion due to electrocardiographic insensitivity in the LCx territory.

The strengths of this study include detailed clinical, laboratory, and angiographic data with a large number of patients with 12 ECG-based NSTEMI and a detailed description of those with sV7-9 STE. In addition, the actual V7-9 was available for nearly three-quarters of the patients, supporting the reliability of the sV7-9 lead findings. This study has several limitations, including its observational, single-center, and retrospective nature. The number of patients with sV7-9 STE was relatively small, leading to low sensitivity. However, the specificity and accuracy were high, and sV7-9 was the sole predictor of LCx STEMI-equivalent ACS. Hence, the clinical utility of sV7-9 remains clear. Finally, the actual V7-9 was ascertained only in selected patients and with delay from synthesized 18-lead ECG. Nevertheless, this unquestionably reflects the limitation of actual V7-9 acquisition, supporting the use of synthesized leads instead.

## 5. Conclusions

Our study demonstrated the potential clinical utility of sV7-9 STE in diagnosing LCx STEMI-equivalent ACS in patients with 12ECG-based NSTEMI. We also observed an association between sV7-9 STE and greater myocardial damage. There was excellent agreement between the synthesized and actual V7-9 findings. Thus, synthesized virtual extra lead findings on admission may help identify patients with NSTEMI requiring primary revascularization.

## Figures and Tables

**Table 1:** Patient characteristics.

	Total *n* = 219	sV7-9 STE *n* = 16	No STE *n* = 203	*p*
*Baseline characteristics*				
Age, years	68.8 (11.9)	63.7 (14.3)	69.2 (11.7)	0.07
Men, *n* (%)	179 (81.7)	12 (75.0)	167 (82.3)	0.70
Body mass index, kg/m^2^	24.2 (22.1, 26.1)	26.1 (23.4, 28.4)	24.1 (22.1, 25.8)	0.07
Current smoking, *n* (%)	72 (32.9)	6 (37.5)	66 (32.5)	0.90
Diabetes, *n* (%)	102 (46.6)	9 (56.2)	93 (46.0)	0.60
Hypertension, *n* (%)	151 (68.9)	13 (81.2)	138 (68.0)	0.41
Dyslipidemia, *n* (%)	113 (51.6)	8 (50.0)	105 (51.7)	1.0
Left ventricular EF, %	57.3 (10.0)	60.0 (8.6)	57.2 (10.2)	0.17
Previous MI, *n* (%)	46 (21.0)	1 (6.2)	45 (22.2)	0.24
Aspirin at baseline, *n* (%)	62 (28.36)	3 (18.8)	59 (29.1)	0.55
Chest symptom on admission, *n* (%)	206 (94.1)	16 (100.0)	190 (93.6)	0.62

*ECG characteristics*				
V1-3 STD, *n* (%)	19 (8.7)	5 (31.2)	14 (6.9)	0.01
V4-6 STD, *n* (%)	63 (28.8)	3 (18.8)	60 (29.6)	0.57
I, aVL STD, *n* (%)	12 (5.5)	0 (0)	12 (5.9)	1.0
II, III, aVF STD, *n* (%)	27 (12.3)	0 (0)	27 (13.3)	0.25
aVR STD, *n* (%)	1 (3.4)	0 (0)	1 (0.5)	1.0
Synthesized V7-9 STD, *n* (%)	10 (4.6)	0 (0)	10 (4.9)	1.0
Atrial fibrillation, *n* (%)	16 (7.3)	0 (0)	16 (7.9)	0.54

*Biochemical analysis*				
hs-cTnI on admission, ng/L	436 (154, 1883)	1055 (380, 6474)	394 (150, 1799)	0.06
NT-proBNP on admission, pg/mL	530 (155, 1829)	263 (150, 1086)	581 (161, 2017)	0.31
Peak hs-cTnI, ng/L	5759 (2060, 19795)	36203 (17255, 93105)	4895 (1913, 15797)	<0.01

*Risk assessment on admission*				
Killip grade, %				0.71
1	195	15 (93.8)	180 (88.7)	
2	16 (7.3)	1 (6.2)	15 (7.4)	
3	8 (3.7)	0 (0)	8 (3.9)	
4	0 (0)	0 (0)	0 (0)	
TIMI score	4 (3, 5)	4 (3, 5)	4 (3, 4)	0.18
GRACE score	144 (119, 172)	113 (94, 142)	145 (122, 173)	0.01

Values are *n* (%) for categorical variables and mean (SD) or median [IQR] for continuous variables. Categorical variables were compared using the chi-square test or Fisher's exact test. Continuous variables were compared using Student's *t*-test or the Mann–Whitney *U* test, accordingly. STE, ST-segment elevation; STD, ST-segment depression; EF, ejection fraction; MI, myocardial infarction; hs-cTnI, cardiac troponin I; NT-pro BNP, N terminal pro B type natriuretic peptide; TIMI, thrombolysis in myocardial infarction; GRACE, Global Registry of Acute Coronary Events.

**Table 2:** Angiographical findings.

	Total *n* = 219	sV7-9 STE *n* = 16	No STE *n* = 203	*p*
*Angiographical findings*				
Culprit vessel, *n* (%)				<0.01
LAD	99 (45.2)	1 (6.2)	98 (48.3)	
LCx	58 (26.4)	13 (81.2)	45 (22.2)	
RCA	62 (28.3)	2 (12.5)	60 (29.6)	

Culprit TIMI flow, *n* (%)				<0.01
0	15 (6.8)	5 (31.3)	10 (4.9)	
1	14 (6.4)	3 (18.8)	11 (5.4)	
2	60 (27.4)	5 (31.3)	55 (27.1)	
3	130 (59.4)	3 (18.8)	127 (62.6)	
Multivessel disease, *n* (%)	61 (27.9)	5 (31.2)	56 (27.6)	0.98
Diameter stenosis, % (AHA class)	90 (90, 990)	100 (99, 100)	90 (90, 99)	0.01
Post-PCI residual stenosis, % (AHA class)	0 [0, 0]	0 (0, 0)	0 (0, 25)	0.77
Post-PCI no reflow, *n* (%)				

(TIMI flow 0–2)	18 (8.2)	0 (0)	18 (8.9)	0.37

Values are *n* (%) for categorical variables and mean (SD) or median [IQR] for continuous variables. Categorical variables were compared using the chi-square test or Fisher's exact test. Continuous variables were compared using Student's *t*-test or the Mann–Whitney *U* test, accordingly. sV7-9, synthesized V7-9; STE, ST-segment elevation; STD, ST-segment depression; LAD, left anterior descending coronary artery; LCx, left circumflex coronary artery; RCA, right coronary artery; AHA, American heart association; TIMI, Thrombolysis in Myocardial Infarction; CK, creatinine kinase; hs-cTnI, cardiac troponin I; PCI, percutaneous coronary intervention.

**Table 3:** Univariate logistic regression analysis and ROC curve analysis for prediction of LCx STEMI-equivalent ACS.

	Univariate logistic regression			ROC curve analysis				
	OR	95% CI	*p* ^*∗∗∗*^	AUC	95% CI	Sensitivity (%)	Specificity (%)	Accuracy (%)
sV7-9 STE	19.0	5.6–63.9	<0.001	0.71	0.58–0.84	46.7	95.6	92.2
V1-3 STD	2.9	0.8–11.5	0.122	0.56	0.45–0.67	20.0	92.6	87.7
Age, years	1.0	1.0–1.0	0.645	0.51	0.34–0.68	46.7	64.7	65.8
Woman	1.7	0.5–5.6	0.388	0.55	0.43–0.66	26.7	82.4	78.5
Left ventricular EF, %	1.0	1.0–1.1	0.475	0.57	0.44–0.71	60.0	61.8	63.5
Killip 1	1.8	0.2–14.2	0.586	0.52	0.45–0.59	93.3	11.3	16.9
GRACE score^*∗*^	1.0	0.8–1.1	0.474	0.56	0.39–0.73	53.3	64.7	63.9
TIMI score	0.64	0.4–1.1	0.077	0.59	0.45–0.74	40.0	68.1	64.4
hs-cTnI on admission^*∗∗*^	1.1	1.0–1.1	0.070	0.65	0.50–0.80	80.0	48.0	60.7

Numbers are calculated based on univariate linear regression and receiver-operation characteristics (ROC) curve analysis. The cut-off point in the ROC curve analysis for calculation of sensitivity, specificity, and accuracy was defined as the value with the highest sum of sensitivity and specificity. Variables included the presence of sV7-9 STE (yes/no), V1-3 STD (yes/no), age (continuous), sex (man or woman), left ventricular ejection fraction (continuous), Killip grade (1 or other), GRACE score (^*∗*^per 10 unit, continuous), and hs-cTnI on admission (^*∗∗*^per 1000 ng/L, continuous). *p*-value threshold was 0.006 after the Bonferroni correction. sV7-9, synthesized V7-9; STE, ST-segment elevation; STD, ST-segment depression; LCx, left circumflex coronary artery; EF, ejection fraction; GRACE, Global Registry of Acute Coronary Events; TIMI, thrombolysis in myocardial infarction; hs-cTnI, cardiac troponin I; OR, odds ratio; CI, confidence interval; ROC, receiver operating characteristics; AUC, the area under the curve.

**Table 4:** Multivariate linear regression analysis for peak hs-cTnI.

Variables	Multivariate linear regression		
	Percent difference (%)	95% CI	*p* ^*∗∗∗*^
sV7-9 STE	308	78–834	0.001
V1-3 STD	115	1–369	0.055
Woman	−5.73	−44.5, 52.2	0.671
GRACE score^*∗*^	3.15	−2.77, 9.42	0.134
hs-cTnI on admission^*∗∗*^	11.1	7.47–14.9	<0.001

Numbers are calculated based on multivariable linear regression. Peak hs-cTnI was logarithmically transformed to approximate the normal distribution, and the exp (coefficient) − 1 was interpreted as the percent difference of the mean hs-cTnI. Models were adjusted for V1-3 STD (yes/no), sex (man or woman), GRACE score (^*∗*^per 10 unit, continuous); and hs-cTnI on admission (^*∗∗*^per 1000 n/L, continuous). *p*-value threshold was 0.006 after the Bonferroni correction. STE, ST-segment elevation; STD, ST-segment depression; GRACE, Global Registry of Acute Coronary Events; hs-cTnI, cardiac troponin I.

## Data Availability

The data used to support the findings of this study are restricted by the Ethics Board of Tsuchiura Kyodo General Hospital to protect patient privacy. Data are available from Tomoki Horie (Contact: tomoki.horie@gmail.com) for researchers who meet the criteria for access to confidential data.

## Abbreviations

ACS: Acute coronary syndrome
ECG: Electrocardiogram
GRACE: Global Registry of Acute Coronary Events
LCx: Left circumflex
NSTEMI: Non-ST-segment elevation myocardial infarction
STEMI: ST-segment elevation myocardial infarction
STD: ST-segment depression
sV7-9 STE: Synthesized V7-9 ST-segment elevation
TIMI: Thrombolysis in myocardial infarction.

## Supplementary Materials

Supplementary Materials Supplemental Table 1: synthesized V7-9 characteristics in patients with LCx-culprit NSTEMI, stratified by LCx TIMI flow of 0 or1 (*n* = 58). Supplemental Table 2: agreement rate of synthesized posterior leads and actual posterior leads for ST-segment morphology (*n* = 164). Supplemental Table 3: diagnostic utility of actual V7-9 STE for the prediction of LCx STEMI-equivalent ACS (*n* = 164). Supplemental Table 4: details of patients with synthesized V7-9 STE.
