# Supplementary material for: Synthesized 18-Lead Electrocardiogram in Diagnosing Posterior Stemi-Equivalent Acute Coronary Syndrome in Patients with NSTEMI
Source: Cardiol Res Pract. 2022 Aug 17;2022:9582174. doi: 10.1155/2022/9582174 (PMC9448611; doi:10.1155/2022/9582174)
Supplement: Supplementary Materials — Supplemental Table 1: synthesized V7-9 characteristics in patients with LCx-culprit NSTEMI, stratified by LCx TIMI flow of 0 or1 (n = 58). Supplemental Table 2: agreement rate of synthesized posterior leads and actual posterior leads for ST-segment morphology (n = 164). Supplemental Table 3: diagnostic utility of actual V7-9 STE for the prediction of LCx STEMI-equivalent ACS (n = 164). Supplemental Table 4: details of patients with synthesized V7-9 STE. [file 9582174.f1.docx]

**Supplemental Table 1. Synthesized V7-9 characteristics in patients with LCx-culprit NSTEMI, stratified by LCx TIMI flow of 0 or1 (n=58)**

|  | **Total**  n=58 | **LCx TIMI 0,1**  n=15 | **LCx TIMI 2,3**  n=43 |
| --- | --- | --- | --- |
| ***Synthesized V7, %*** |  |  |  |
| No change | 28 (48.3) | 5 (33.3) | 23 (53.5) |
| STE | 11 (19.0) | 6 (40.0) | 5 (11.6) |
| STD | 5 (8.6) | 1 (6.7) | 4 (9.3) |
| Inverted T wave | 14 (24.1) | 3 (20.0) | 11 (25.6) |
| ***Synthesized V8, %*** |  |  |  |
| No change | 26 (44.8) | 4 (26.7) | 22 (51.2) |
| STE | 13 (22.4) | 7 (46.7) | 6 (14.0) |
| STD | 5 (8.6) | 1 (6.7) | 4 (9.3) |
| Inverted T wave | 14 (24.1) | 3 (20.0) | 11 (25.6) |
| ***Synthesized V9, %*** |  |  |  |
| No change | 27 (46.6) | 4 (26.7) | 22 (51.2) |
| STE | 12 (20.7) | 7 (46.7) | 6 (14.0) |
| STD | 5 (8.6) | 1 (6.7) | 4 (9.3) |
| Inverted T wave | 14 (24.1) | 3 (20.0) | 11 (25.6) |

Values are n (%).

Abbreviations: LCx, left circumflex coronary artery; STE, ST-segment elevation; STD, ST-segment depression; TIMI, Thrombolysis in Myocardial Infarction.

**Supplemental Table 2. Agreement rate of synthesized posterior leads and actual posterior leads for ST-segment morphology (n=164)**

|  | **No change** | **STE** | **STD** | **Inverted T wave** |
| --- | --- | --- | --- | --- |
| Actual V7-9 | 105 | 13 | 9 | 37 |
| Synthesized V7-9 | 93 | 14 | 10 | 47 |

Values are n.

Abbreviations: STE, ST-segment elevation; STD, ST-segment depression.

**Supplemental Table 3. Diagnostic utility of actual V7-9 STE for the prediction of LCx STEMI-equivalent ACS (n=164)**

|  | **Univariate logistic regression** | | | **ROC curve analysis** | | | | |  |  | |
| --- | --- | --- | --- | --- | --- | --- | --- | --- | --- | --- | --- |
|  | **OR** | **95%CI** | **P***** | **AUC** | **95%CI** | **Sensitivity (%)** | **Specificity (%)** | **Accuracy (%)** | **P** | |  |
| Actual V7-9 STE | 12.9 | 3.3-49.6 | <0.001 | 0.68 | 0.54-0.83 | 50.0 | 94.7 | 91.5 | 0.317 | |  |
| sV7-9 STE | 18.0 | 4.7-68.5 | <0.001 | 0.72 | 0.57-0.87 | 50.0 | 94.7 | 91.5 |  | |  |

Numbers are calculated based on univariate linear regression and receiver-operation characteristics (ROC) curve analysis.

The cut-off point in the ROC curve analysis for calculation of sensitivity, specificity and accuracy were defined as the value with the highest sum of sensitivity and specificity. DeLong’s test for two correlated ROC curves was used to assess the difference in AUC of the two ROC curve analysis.

Variables included the presence of actual V7-9 STE (yes/no) and sV7-9 STE (yes/no).

P-value threshold was 0.025 after Bonferroni correction.

Abbreviations: sV7-9, synthesized V7-9; STE, ST-segment elevation; OR, odds ratio; CI, confidence interval; ROC, receiver-operating characteristics; AUC, area under curve.

**Supplemental Table 4. Details of patients with synthesized V7-9 STE**

| ***Age (years)/ Sex*** | ***Previous MI*** | ***V1-3 STD*** | ***Admission***  ***hs-cTnI (ng/L)*** | ***GRACE score*** | ***True V7-9*** | ***CulpritVessel*** | ***MVD*** | ***Culprit TIMI flow*** | ***Peak***  ***hs-cTnI (ng/L)*** | ***MRI findings*** |
| --- | --- | --- | --- | --- | --- | --- | --- | --- | --- | --- |
| 60/man | (-) | (-) | 152 | 131 | STE | LAD | (-) | 3 | 554 | No LGE |
| 74/woman | (-) | (-) | 470 | 153 | STE | LCx | (-) | 2 | 2196 | No LGE |
| 73/man | (-) | (+) | 170 | 144 | STE | LCx | (-) | 3 | 10371 | NA |
| 88/man | (+) | (+) | 4854 | 233 | STE | RCA | (+) | 3 | 16495 | NA |
| 68/woman | (-) | (-) | 6556 | 86 | NA | LCx | (-) | 2 | 19535 | Posterior LGE |
| 82/man | (-) | (+) | 10647 | 187 | STE | LCx | (-) | 1 | 24249 | NA |
| 45/man | (-) | (-) | 1373 | 71 | STE | LCx | (-) | 2 | 25972 | Posterior LGE |
| 44/woman | (-) | (+) | 737 | 56 | STE | LCx | (-) | 2 | 31058 | NA |
| 48/woman | (-) | (-) | 182 | 82 | No change | LCx | (-) | 1 | 41348 | NA |
| 72/man | (-) | (-) | 25762 | 141 | NA | RCA | (+) | 0 | 49298 | NA |
| 67/man | (-) | (-) | 610 | 96 | STE | LCx | (+) | 2 | 58844 | Posterior LGE |
| 80/man | (-) | (-) | 1911 | 132 | STE | LCx | (-) | 0 | 88624 | NA |
| 59/man | (-) | (-) | 16419 | 103 | STE | LCx | (+) | 0 | 94598 | Posterior LGE |
| 65/man | (-) | (-) | 6228 | 108 | STE | LCx | (-) | 0 | 107823 | Posterior LGE |
| 44/man | (-) | (+) | 350 | 118 | NA | LCx | (-) | 0 | 125730 | Posterior LGE |
| 50/man | (-) | (+) | 488 | 104 | STE | LCx | (+) | 1 | 126398 | Posterior LGE |

Abbreviations: MI, myocardial infarction; STE, ST-segment elevation; STD, ST-segment depression; LAD, left anterior descending coronary artery; LCx, left circumflex coronary artery; RCA, right coronary artery; MVD, multivessel disease; GRACE, Global Registry of Acute Coronary Events; TIMI, Thrombolysis in Myocardial Infarction; hs-cTnI, cardiac troponin I; MRI, magnetic resonance imaging; LGE, late gadolinium enhancement; NA, not available
